# Performance of a solar photocatalysis reactor as pretreatment for wastewater via UV, UV/TiO_2_, and UV/H_2_O_2_ to control membrane fouling

**DOI:** 10.1038/s41598-022-20984-0

**Published:** 2022-10-06

**Authors:** Nisreen S. Ali, Khairi R. Kalash, Amer N. Ahmed, Talib M. Albayati

**Affiliations:** 1 Materials Engineering Department, College of Engineering, Mustansiriyah University, Baghdad, Iraq; 2 Environment and Water Directorate, Ministry of Science and Technology, Baghdad, Iraq; 3 Department of Chemical Engineering, University of Technology- Iraq, 52 Alsinaa St., PO Box 35010, Baghdad, Iraq

**Keywords:** Chemical engineering, Environmental sciences, Materials science

## Abstract

The performance of a solar photocatalysis reactor as pretreatment for the removal of total organic carbon (TOC) and turbidity from municipal wastewater was achieved by implementing an integrated system as tertiary treatment. The process consisted of ultraviolet (UV) sunlight, UV sunlight/H_2_O_2_, and UV sunlight/TiO_2_ nanocatalysts as pretreatment steps to prevent ultrafiltration (UF) membrane fouling. The characterization of TiO_2_ was conducted with X-ray diffraction spectroscopy, Fourier-transform infrared spectroscopy, scanning electron microscopy , and Brunauer–Emmett–Teller surface area analysis. This study investigated the effect of time and solar radiation using UV, UV/H_2_O_2_, and UV/TiO_2_ to remove TOC and turbidity. The transmembrane pressure improvement was studied using a UF membrane system to pretreat wastewater with different UV doses of sunlight for 5 h and UV/H_2_O_2_ and UV/TiO_2_. The results showed that the highest removal efficiency of the turbidity and TOC reached 95% and 31%, respectively. The highest removal efficiency of the turbidity reached 40, 75, and 95% using UV, UV/H_2_O_2_, and UV/TiO_2_, respectively, while the optimal removal efficiency of TOC reached 20%, 30%, and 50%, respectively.

## Introduction

Wastewater treatment plants use activated sludge systems as a secondary treatment to remove organics, suspended solids, and nutrients^1^; membrane filtrations are conducted as a tertiary treatment to produce high-quality water for reuse and reclamation for various purposes^2^. The most advanced wastewater plants employ tertiary treatments based on membrane technologies, which mainly consist of pressure-driven membrane-like ultrafiltration (UF) and reverse osmosis (RO)^3–9^. Membrane technologies, particularly pressure-driven membranes, are considered the most promising approaches for reusing water^9^. However, microorganism colloids, dissolved organic matter, and suspended solids in the wastewater effluent.

cause membrane fouling on the surface or within the membranes’ pores. Approximately 10% of the effluent dissolved organic carbon (DOC) contributes to membrane fouling^10,11^, which decreases the membrane performance productivity, increases backwashing, and increases the costs of both membrane replacement and general treatment^12^. Decreasing fouling is of fundamental concern in membrane processes because it can increase the membranes’ operational life and decrease the membrane cleaning operations^13^. Numerous approaches, such as adsorption^14–17^, coagulation^18,19^, oxidation^20^, and ionic exchange^21^, have been investigated to reduce organic fouling, prevent fouling on the membranes, and improve the membrane’s filtration performance. Among these, advanced oxidation processes (AOPs) are some of the most promising^22^. The destructive process of AOPs is highly effective at removing organic compounds, especially from water. AOPs are characterized by the generation of highly reactive species, such as hydroxyl radicals (OH), which have a very high redox potential (2.8 V)^21–24^. Advanced oxidation processes can consist of chemical processes, including ozonation, H_2_O_2_ oxidation, the Fenton reaction, electrochemical or photochemical oxidation, and photochemical processes (e.g., photocatalysis, photolysis, the photo-Fenton reaction, solar heterogeneous photocatalytic oxidation, and combined UV/TiO_2_/O_3_ and UV/O_3_)^24,25^. Simultaneously using two or more types of AOPs has proven more effective in removing organic pollutants than using a single method alone^24,26,27^. Because of its nontoxicity, physical and optical properties, high stability, and high photocatalytic activity, titanium dioxide (TiO_2_) is the most commonly used and investigated catalyst^25^. It has numerous ideal properties (e.g., eco-friendly, low energy bandgap, resistance to photo-corrosion, and high UV absorption) and can be used without additives^26^. Further, TiO_2_ can only operate in the UV spectrum^27–29^. Advantageously, solar heterogeneous photocatalytic oxidation does not rely on lamps or LEDs^29–31^. This wavelength is controlled by the bandgap of the photocatalyst, which produces hydroxyl radicals and holes. The most frequently investigated photocatalyst, TiO_2_, exhibits a bandgap of 3.0 eV for the rutile modification and 3.2 eV for the anatase modification. The TiO_2_ material can only absorb wavelengths below 400 nm. Approximately 5% of solar radiation comes from this spectral range^29,32–34^. TiO_2_ particles entrapped in membranes or having titanium deposition on their surfaces may exhibit improved hydrophilicity, thereby reducing fouling^30,31^.

In this research, a commercial powder catalyst of TiO_2_ was employed and characterized for the photocatalytic degradation of municipal contaminants by adding UV/H_2_O_2_. The structure and performance of TiO_2_ were determined by various characterizations using scanning electron microscopy (SEM), X-ray diffraction (XRD), Brunauer–Emmett–Teller (BET) surface area analysis, and Fourier-transform infrared spectra (FT-IR). The study aimed to evaluate the feasibility and efficiency of using UV, UV/H_2_O_2_, and UV/TiO_2_ as a pretreatment step to control fouling in UF membranes while using the solar photooxidation process for the tertiary treatment of secondary effluent from municipal wastewater treatment plants.

## Materials and methods

### Materials

During the present study, wastewater from a wastewater treatment plant in Baghdad, Iraq, was collected and analyzed. Titanium dioxide (TiO_2,_ purity ≥ 99%) and hydrogen peroxide (H_2_O_2,_ 30% solution [w/w] in H_2_O) were used, with all chemicals purchased from Thomas Baker (India).

### Characterization

X-ray powder diffraction (XRD) tests were conducted with a diffraction unit (Shimadzu-6000, Japan) at the Nanotechnology and Advanced Research Materials Center/University of Technology (Baghdad). A scanning electron microscope (SEM) (VEGA 3 LM, Germany) available at the Central Service Laboratory (College of Education for Pure Sciences/Ibn Al Haitham/Baghdad University) was used to perform morphological analysis of the catalyst TiO_2_. The total pore volume and specific surface area of the catalyst TiO_2_ were measured utilizing a Brunauer–Emmett–Teller (BET) surface area analyzer (Q-surf 9600, USA) from the Petroleum Research and Development Center (Baghdad). A Fourier-transform infrared (FT-IR) spectrophotometer (Bruker Tensor 27, Germany) recorded the FT-IR spectra ranging from 500 to 4000 cm^−1^.

### Pretreatment process setup

**Figure 1:**
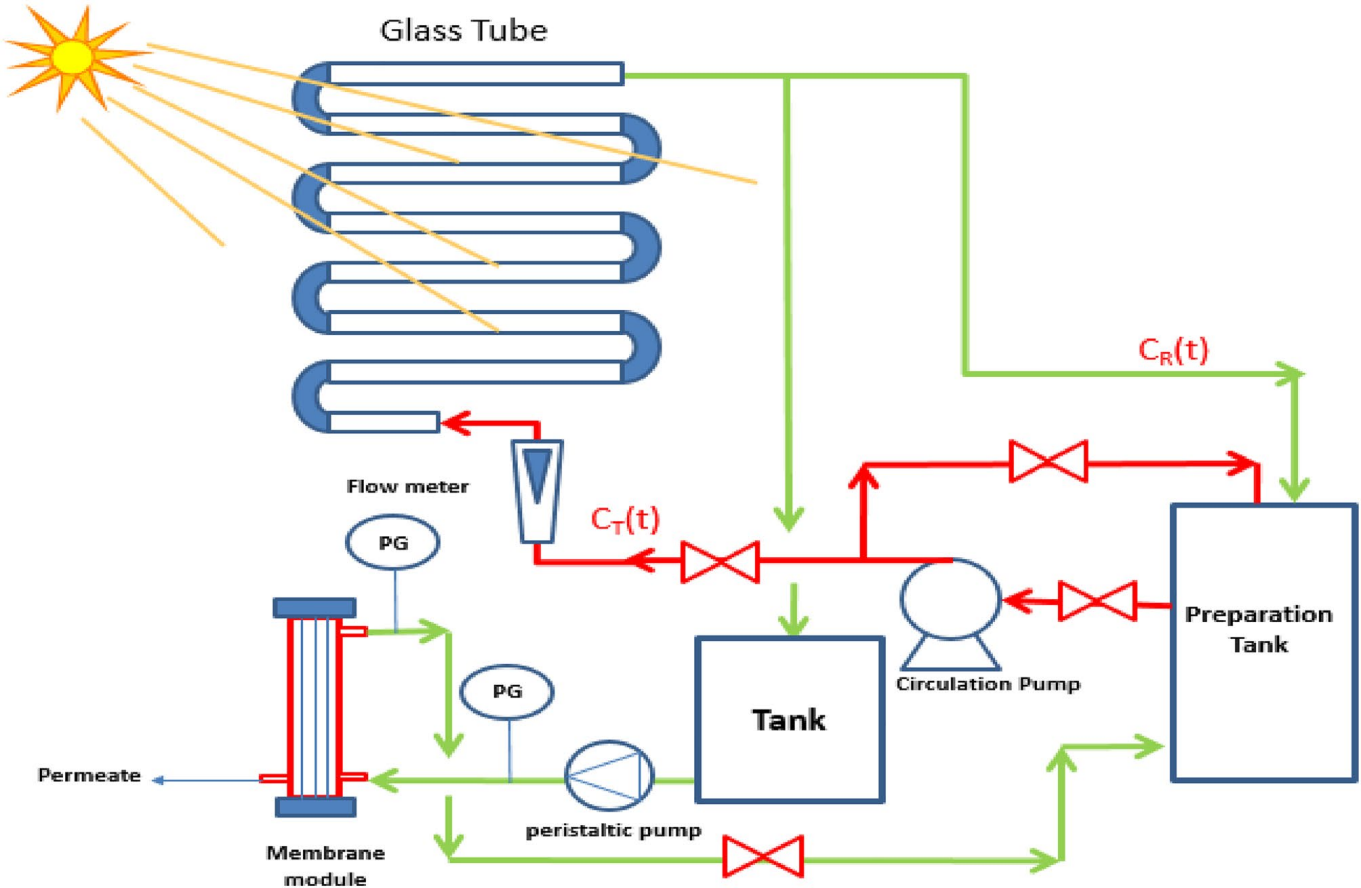
Schematic representation of the solar photocatalytic reactor and membrane filtration systems.

The solar photooxidation process of wastewater pretreatments was carried out with sunlight using a solar reactor system consisting of eight connected tubular glass pipes (0.022 m inside diameter and 0.55 m long). These pipes were supported with steel construction and sheltered via a reflective surface constructed from aluminum foil, as shown in Fig. 1. To mix and circulate the water at different flow rates; the photo-reactor was equipped with a pump with a capacity of 10–100 mL/min to ensure wastewater homogeneity in the glass tubes. The photo reactor was mounted and tilted at a 45° angle.

### Ultrafiltration system

The experimental arrangement of the ultrafiltration system, shown in Fig. 1, involved a membranes module, pressure gauge, and peristaltic pump. The UF module was connected to an influent tank with 5 L of wastewater collected from the photo-reactor tank. The peristaltic pump was operated at different flow rates. The pressure gauge continuously measured the transmembrane pressure (TMP).

### Effluent wastewater

The wastewater sample was collected and analyzed from a wastewater treatment plant in Baghdad city. It was taken from the secondary clarifiers without nutrient removal and saved at 4 °C in a refrigerator throughout this study. Table 1 shows the physicochemical properties of wastewater influent used in this experiment without any other pretreatment.

**Table 1:** Main Characteristics of the secondary wastewater.

No	Parameter	Unit	Value
1	BOD_5_	mg/L	15–20
2	COD	mg/L	10–20
3	NTU	Mg/L	5–12
4	NH_3_-N	mg/L	4–5
5	TOC	mg/L	13–16
6	NO_2_-N	mg/L	0.6–0.8
7	TN	mg/L	33–36
8	PO4_3_^−3^	mg/L	18–20
9	TDS	mg/L	1200
10	pH	–	7–7.5

All experiments were conducted in two parts. First, wastewater was pretreated using a solar reactor with UV sunlight, UV sunlight/H_2_O_2_, and UV sunlight/TiO_2_ at a constant flow rate of 50 mL/min for 6 h. At 30-min intervals, the transmembrane pressure (TMP) was recorded to indicate membrane fouling. At the same intervals, the feed and permeate samples were also analyzed. The initial H2O2 concentration in UV sunlight/H2O2 experiments was 15 mg/L. This H2O2 concentration was carefully chosen according to the typical range (5–50 mg/L) used in other works related to the UV/H2O2 treatment indicated by Zhang et al.^6^. On the other hand, UV sunlight/TiO2, with a catalyst concentration of 0.75 g/L, was used in our experiments. Based on the results indicated by Ghaly et al.^33^.

Second, a cleaning process was conducted at the end of the experimental run. According to the subsequent cleaning procedure, the membrane was aerated with air bubbles for 20 min to remove most of the cake layer. After that, a 0.5 g/L surfactant solution was prepared, and the membrane was soaked, followed by bleach cleaning.

### Analytical methods

UV_254_ absorption was measured using a Shimadzu UV visible spectrophotometer as an indicator of the total organic carbon (TOC). The measurement of the UV radiation was conducted at the “Center of Solar Energy Research—Ministry of Science and Technology” using Davis 6152 C Vantage Pro 2 Weather Station radiometer. The following equation was used to calculate the percentage removal of wastewater^16,35–39^:

1 $${\text{\% }}Removal{\ }of{ }wastewater = \frac{{\left( {Ci - Co} \right)}}{Ci}{*}100$$

where C_i_ = initial concentration of wastewater, and C_o_ = final concentration.

## Results and discussion

### Characterization of the catalyst

**Figure 2:**
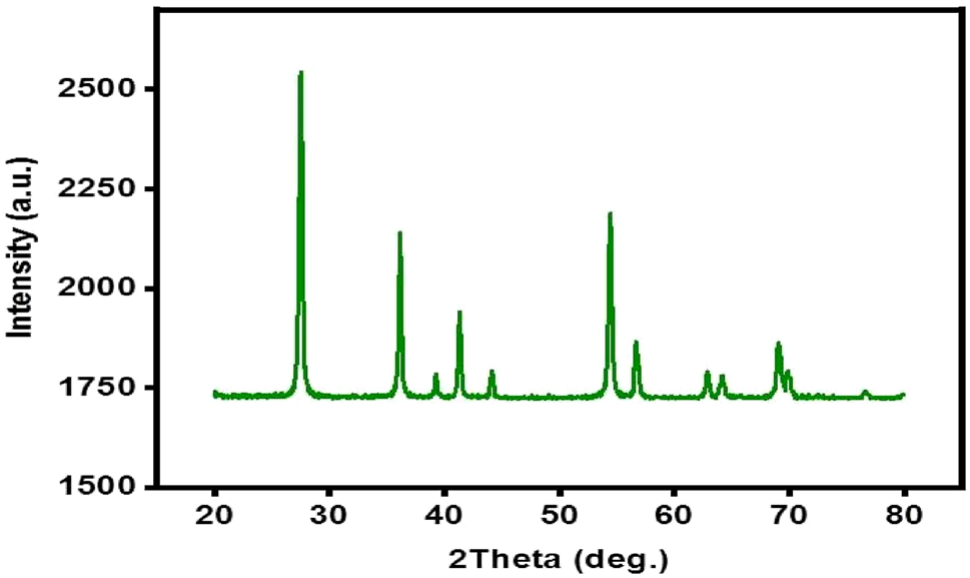
XRD images of TiO_2_.

**Figure 3:**
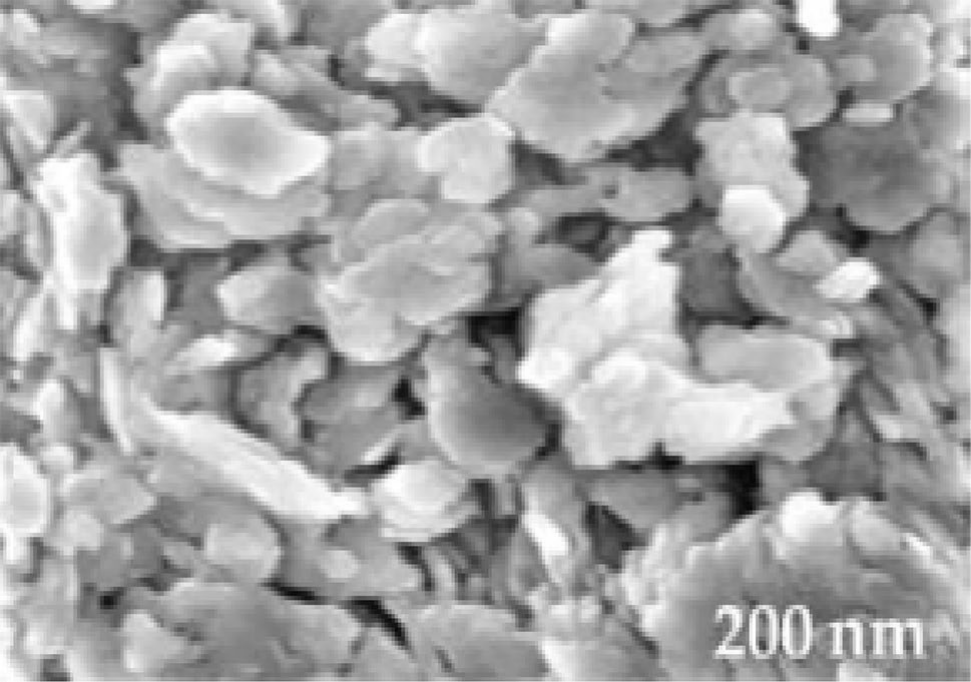
SEM images of TiO_2_.

**Figure 4:**
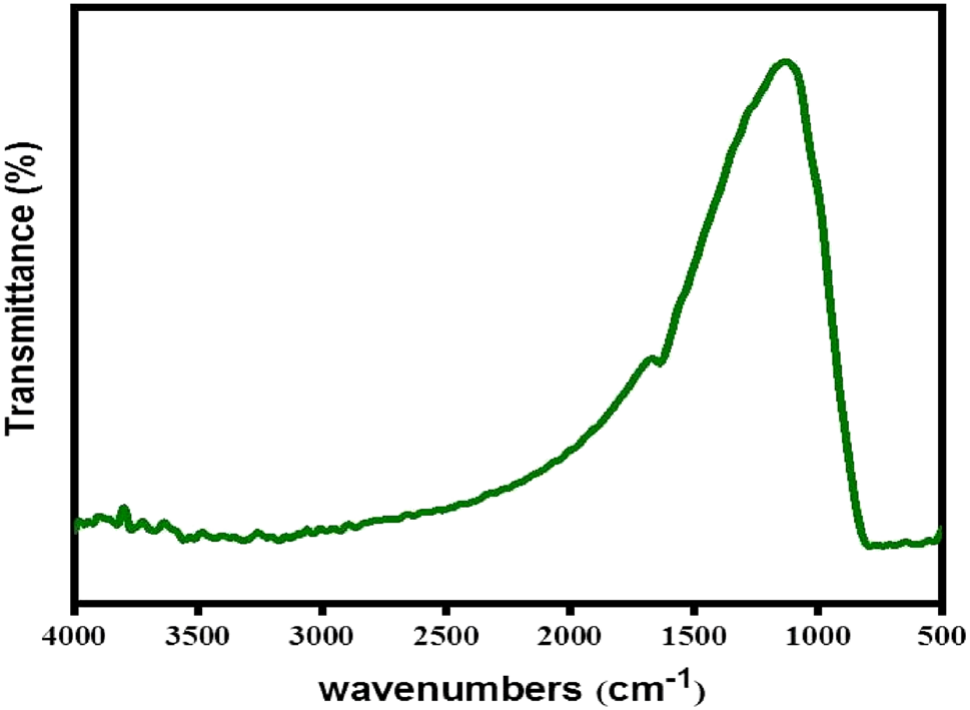
FTIR images of TiO_2_.

Figure 2 displays the spectroscopic structures of TiO_2_ that were analyzed by X-ray diffraction (XRD). The crystal planes [(101), (004), (200), (105), (211), and (204)] appeared in the powder catalyst of TiO_2_^31,40^. The general morphologies and microstructures of TiO_2_ were investigated by SEM analysis, as shown in Fig. 3. The surface morphology of TiO_2_ is also displayed in Fig. 3. Spherical nanoparticles with diameters mainly ranging from 14 to 20 nm were revealed in the SEM image of TiO_2_ in Fig. 3, echoing the observations of TiO_2_ morphology reported by Jin et al.^36^. However, the results of Chong et al. agree with these conclusions^41^. Brunauer investigated the pore volume and surface area–Emmett–Teller (BET) surface area analysis to understand the roles of TiO_2_. The specific surface area (S_BET_) of the TiO_2_ was 290 m^2^/g, and the total pore volume was 0.64 cm^3^/g; the homogeneous distribution of nano-TiO_2_ particles and the unique creation of a kaolin-layered structure could explain the huge surface area and total pore volume of the TiO_2_. The FT-IR spectra of the samples, shown in Fig. 4, were used to analyze the vibrational bands and interface interactions. The range of 699–732 cm^−1^, representing the obvious stretching vibration of Ti–O–Ti, was displayed by all three samples^42^, while the stretching vibration of the hydroxyl bonds appeared on the region of the broad peaks within the range from 3100 to 3600 cm^−1^. Due to the surface-adsorbed water molecules, an H–O–H bending vibration can be assigned at the peak of 1630 cm^−1^. Hydroxyl bonds cause improved photocatalytic activity through the adsorbed water molecules and lead to the formation of the hydroxyl radical (OH^•^), which can be classified as an oxidant reacting with oxygen (O_2_) or a photo-induced hole (h^+^)^42^.

### Influence of radiant flux

**Figure 5:**
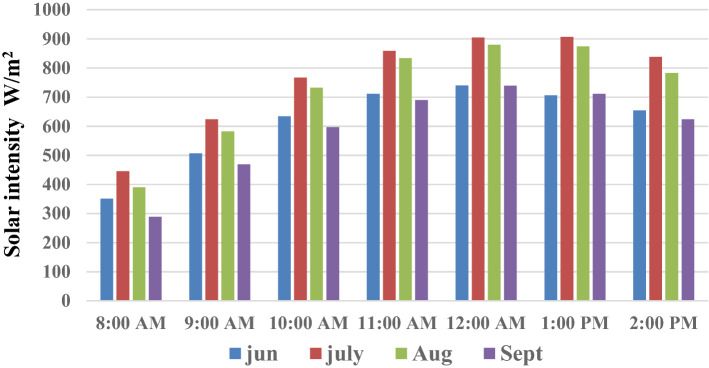
The variation of solar intensity with the experimental time at several months.

The chosen UV dosages were obtained from natural solar irradiation. The time of illumination vs. solar intensity is plotted in Fig. 5. All experiments were conducted between 8 a.m. and 2 p.m. local time, at a mean irradiance of 763 W/m^2^. The mean UV intensity for the complete experiment changed depending on the solar intensity. The UV intensity was recorded between 14.5 and 15.66 W/m^2^ from 8:00 a.m. to 2:00 p.m., corresponding to 2% of the power of the solar irradiation. The maximum solar intensity was 900 W/m^2^ at noon, with a UV intensity of 17.6 W/m^2^. In most common cases for disinfection, UV dosages do not exceed the value of 0.5 J cm^−2^, but the UV dosages obtained from natural solar irradiation were relatively higher than 0.5 J cm^−2^^43^.

### Fouling reduction using UV-based pretreatment

**Figure 6:**
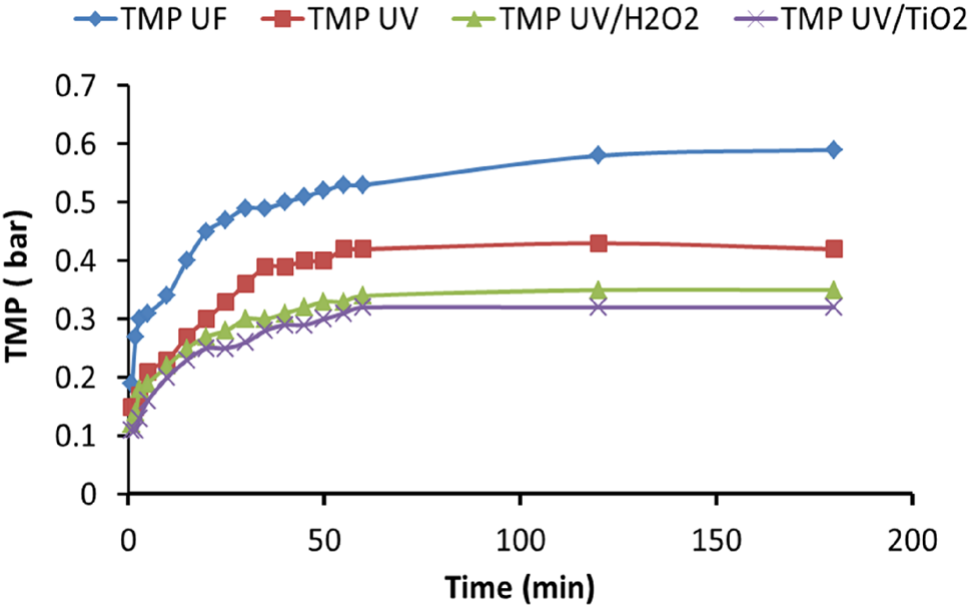
The effect of the time on TMP by UF, UV, UV/H_2_O_2_, and UV/TiO_2_.

**Figure ****6** shows the transmembrane pressure (TMP) improvement through the ultrafiltration system’s pretreated water using different UV intensity doses from the solar irradiation over 6 h. Through the first 60 min, the transmembrane pressure increased rapidly after filtration and reached a maximum after 6 h, after which the TMP decreased. However, without pretreatment, the TMP was at its highest, recording around 0.59 bar at the end of the run. In contrast, at the end of the run where UV sunlight was used, the reach was lower and reduced, indicating that pretreatment with UV sunlight dosages had a positive effect, yielding a TMP of approximately 0.42 bar. After pretreating the water with UV sunlight/H_2_O_2_ with an initial H_2_O_2_ concentration of 15 mg/L, the TMP was around 0.35 bar. In a simultaneous solar irradiation experiment, the TMP was recorded at approximately 0.32 bar at a catalyst concentration of 0.75 g/L. The reduction in the TMP in all previous experiments in this study has a similar trend.

**Figure 7:**
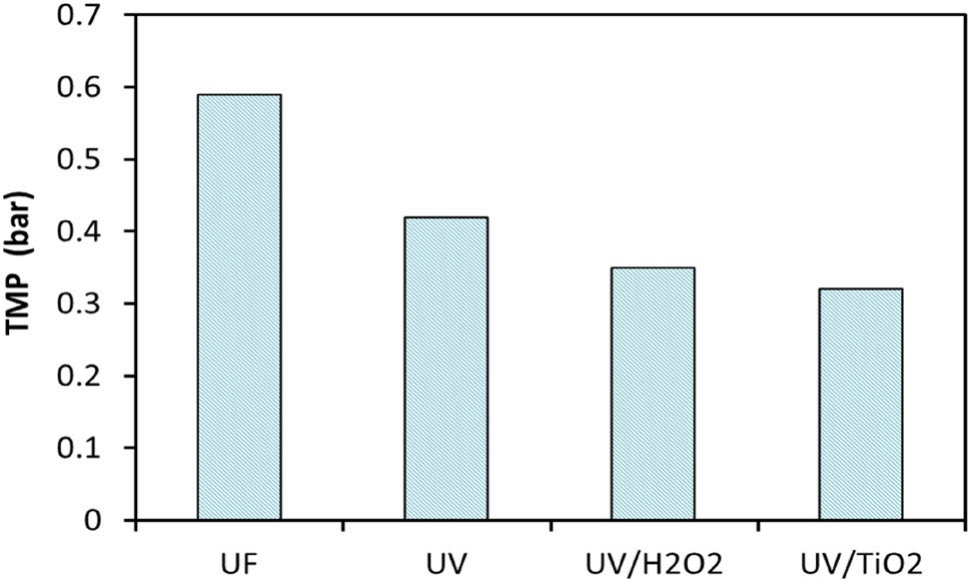
The effect of using UF, UV, UV/H_2_O_2_, and UV/TiO_2_ on TMP.

Figure 7 shows that the maximum TMP peaked after 6 h using the UF system with the water treated with or without UV sunlight. It can be observed that the maximum TMP found in all experiments with UV sunlight, UV sunlight /H_2_O_2_, and UV sunlight/TiO_2_ was reduced by about 29, 41, and 45.8%, respectively, compared with those without pretreatment^43^.

### UV sunlight, UV sun/H_2_O_2,_ and UV sun/TiO_2_ as pretreatments to enhance water quality

#### Removal efficiency of turbidity and TOC using the ultrafiltration (UF) process

**Figure 8:**
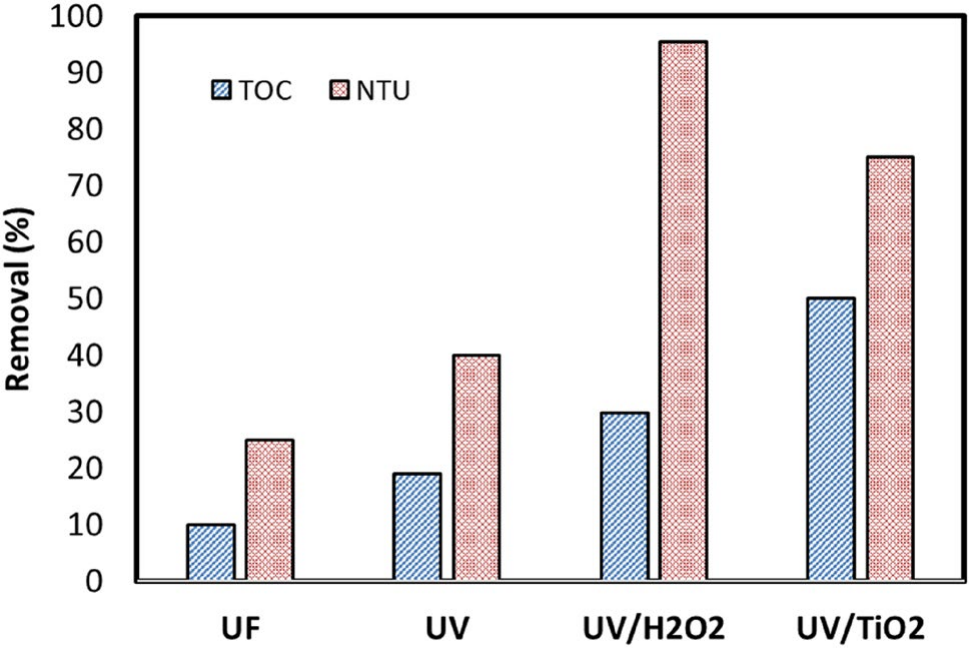
The effect of using UF, UV, UV/H_2_O_2_, and UV/TiO_2_ on removal efficiency of TOC and turbidity.

The removal efficiency was reduced by 25%, with an initial value of 8 NTU and a final value of 6 NTU, while the removal efficiency for TOC was about 10%, with an initial value of 14 mg/L and a final value of 12.6 mg/L as shown in Fig. 8.

#### Removal efficiency of TOC and turbidity after the (UF) process using UV sunlight

The removal efficiency was increased up to 20%. It enhanced the removal efficiency of the turbidity to 40% as shown in Fig. 8. The results obtained appeared that UV irradiation is an effective technology for the removal of total organic carbon (TOC) and turbidity from municipal wastewater during the post-treatment of secondary effluents. Still, its efficiency depends on the type of organic compound and secondary effluent quality. In general, no influence of the kind of effluent was noticed for organic compounds with very slow or fast photo transformation kinetics. In contrast, for those compounds with intermediate kinetics, their photo transformation would be enhanced in effluents of better quality. Therefore, despite UV treatment being an efficient technology to photo transform organic compounds, small development or modifications such as; increasing UV dose, using oxidant agents such as; H_2_O_2_, and using catalysts to enhance the reduction of total organic carbon (TOC) and turbidity in UV systems^44^.

#### Removal efficiency of TOC and turbidity after the (UF) process Using UV sunlight/H_2_O_2_

The turbidity removal efficiency after UF improved by about 95%; in the experiments using UV sunlight/H_2_O_2_ at an H_2_O_2_ concentration of 15 mg/L within a 6-h period of solar irradiation, and the TOC removal efficiency increased up to 30%, as shown in Fig. 8.

The higher TOC removal in the UV/H_2_O_2_ pretreatment process can be explained by the fact that the ⋅OH radicals generated by the process are highly reactive and oxidize the organic substances^45^. The higher removal efficiency of the turbidity of the pretreated wastewater was associated with the UV or UV/H_2_O_2_ pretreatments affecting the TOC and likely affecting the suspended solids’ size.

#### Removal efficiency of TOC and turbidity after the (UF) process Using UV sunlight/TiO_2_

In the case of using TiO_2_ with UV sunlight, degradation experiments require a specific amount of catalyst, so the optimum catalyst loading for removing TOC from wastewater must be determined to avoid using the excess catalyst. Several authors have investigated the photocatalytic oxidation process as a function of catalyst loading with different semiconductor copmpounds^33,46,47^. Since TiO_2_ scatters light, excess TiO_2_ in suspensions will prevent sunlight from penetrating^47,48^. Therefore, the dosage of TiO_2_ in the photoreactor needs to be optimized, resulting in lower photocatalyst costs. Based on the results above, the optimum TiO2 concentration in this study was 0.75 g/L, the exact dosage of catalysts used by Ghaly et al.^33^ in their experiments.

In a simultaneous solar irradiation experiment, the TOC was removed from the wastewater by 50% at a catalyst concentration of 0.75 g/L. According to the results, the high decomposition observed under both solar light and TiO_2_ was solely due to the photocatalytic reaction of the semiconductor particles. The wastewater degradation was induced by the photoexcitation of semiconductors to electron–hole pairs on the catalyst’s surface^49^. At an optimum TiO_2_ loading of 0.75 g/L, the photocatalytic oxidation of the treated wastewater also showed a removal efficiency of 87.5% NTU, as shown in Fig. 8.

### The removal efficiency of TOC as a function of solar UV intensity

**Figure 9:**
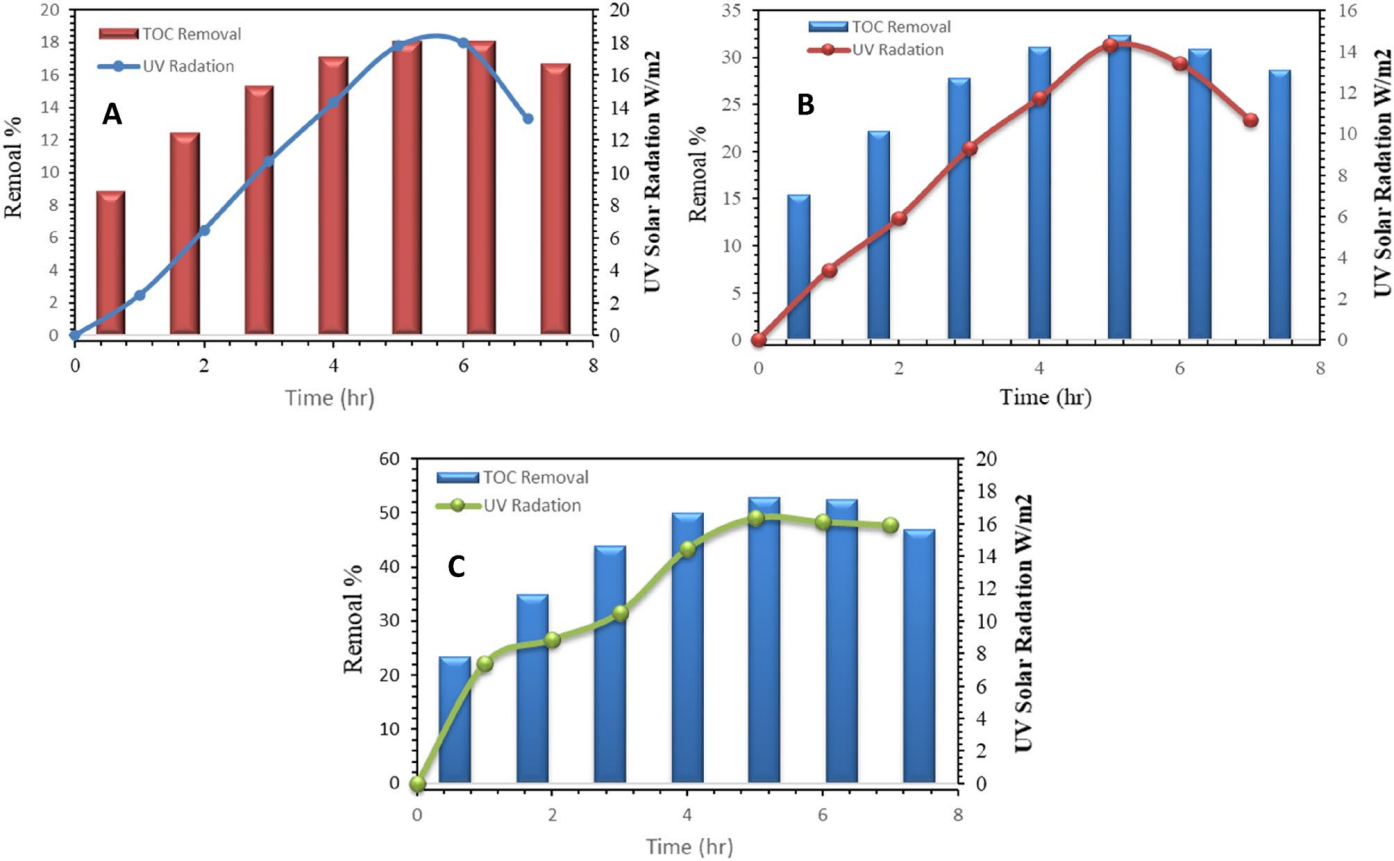
The effect of time and UV intensity on removal efficiency of TOC by using UF,(**A**): UV, (**B**): UV/H_2_O_2,_ and (**C**): UV/TiO_2_.

The removal efficiency increased as the solar UV intensity increased in all experiments. Figure 9 shows that the TOC content decreased gradually as the UV intensity increased. In the case of UV sunlight alone, the removal of TOC increased and caused removals of from 2.49 to 18%, with UV intensity between 8.9 and 18.14 W/m^2^. Simultaneous UV intensity caused 7.4 to 31.3% TOC removal at an H_2_O_2_ concentration of 15 mg/L within a 6-h period of solar irradiation. Instantaneous solar irradiation with a UV intensity between 7.8 and 15.66 W/m^2^ caused 50% TOC removal at a catalyst dosage of 0.75 g/L of TiO_2_ within 6 h of solar irradiation. According to the results, high decomposition under both UV intensity from solar light and TiO_2_ processes was exclusively due to the photocatalytic reaction of the semiconductor particles. Furthermore, these experiments showed that UV intensity and TiO_2_ were necessary to treat wastewater effectively^49^.

### Comparative study

This study dealt with treating municipal wastewater to remove TOC and turbidity by using UV, UV/H_2_O_2_, and UV/TiO_2_ in the solar photooxidation process through a batch system and using ultrafiltration membranes to control the membrane fouling process. Table 2 compares this study and others for the removal of TOC and turbidity. This table presents that the performance of a solar photocatalysis reactor as pretreatment for wastewater in an integrated system was a promising process for removing total organic carbon (TOC) and turbidity from municipal wastewater by implementing an integrated system as tertiary treatment.

**Table 2:** Comparison between this study and other studies.

No.	Process	Removal	Process conditions	References
1	Microfiltration and visible-light-driven photocatalysison g-C3N4nanosheet/reduced graphene oxide membrane	TOC = 21 %, Turbidity = 84% by memberance TOC= 42%, Turbidity = 90% by integrated process	Time = 1 h. For the g-C_3_N_4_NS/RGO/CA (100 gm in 200 mL) membrane under visible light irradiation, the stable permeation flux = 140 L m^−2^h^−1^, pH = 7.1.	^50^
2	Solar photocatalysis using TiO2/ZnO/H2O2 to pretreat reverse osmosis (membrane fouling)	TOC = 76.5%	Reaction time = 179 min, TiO2 =,0.51 g/L, ZnO = 0.46 g/L, pH= 6.9 and H2O2 = 0.89 mL/L	^51^
3	Photocatalytic by bi-polymer electrospun nanofibers embedding Ag_3_PO_4_/P25 composite	TOC = 86 %, turbidity = 50%	Reaction time = 150 min, flow rate (5 mL/h), pH = 7	^52^
4	photocatalysis, Fenton-based processes and ozonation	TOC < 20%, TOC = 74% by ozonation combined with H_2_O_2_	Reaction time = 4 h, H_2_O_2_/Fe = 0.5O_3_= 4 g h^−1^ , H_2_O_2_ = 1500 mg L^−1^ , pH =10, reaction time = 2 h , temperature= 20 °C	^53^
5	hybrid ultrafiltration/ reverse osmosis (UF/RO) systeme	TOC = 98%	TMP = 3 bar, CFV =1 m/s, temperature = 40 °C, and pH = 9	^54^
6	Solar photocatalysis reactor by UV, UV/H_2_O_2_, and UV/TiO_2_ and ultrafiltration membrane fouling	By UV TOC = 20%, Turbidity = 40% by UV/H_2_O_2_ TOC= 30%, Turbidity= 95% by UV/TiO_2_ TOC = 50%, Turbidity = 87.8% by	Reaction time of 6 hr., solar radiation of 14.8–18.4 w/m^2^.hr, pH = 7.0, H_2_O_2_ = 15 mg/L, and flow rate = 50 mL/min	This study

## Conclusions

This solar photooxidation process and membrane filtration system study investigated the efficiency and performance evaluation for TOC and turbidity removal from municipal wastewater at a reaction time of 6 h. Using a solar photooxidation process, experiments were conducted using UV, UV/H_2_O_2_, and UV/TiO_2_. The UV intensity of 18.41 w/m^2^.hr achieved its highest reduction of TOC and turbidity, which was 20 and 40%, respectively. The UV intensity of 14.8 w/m^2^.hr with a 15 mg/L concentration of H_2_O_2_ at a pH of 7.0 achieved its highest reduction in the TOC and turbidity, 30 and 95%, respectively. The UV intensity of 17.6 w/m^2^.hr with a catalyst concentration of 0.75 g/L of TiO_2_ achieved its highest reduction of TOC and turbidity, 50 and 87.8%, respectively. In the membrane fouling process using ultrafiltration, the TMP with ultrafiltration combined with UV, UV/H_2_O_2,_ and UV/TiO_2_ versus ultrafiltration alone was reduced by about 29.41, 41, and 45.8%, respectively, after 6 h, with a constant flow rate of 50 mL/min, with the highest removal of TOC and turbidity being 50% and 95%, respectively. It might be concluded from this study that the processes of UV, UV/H_2_O_2_, and UV/TiO_2_ using the solar photooxidation process prevented the UF membrane fouling with higher removal of TOC and turbidity.

## Data Availability

All data generated or analysed during this study are included in this published article.
